# Rates and predictors of hospital visits after “Possible AF” alerts from a home ECG monitor in older adults with hypertension: A sub-analysis of the Omron Heart Study

**DOI:** 10.1371/journal.pone.0353867

**Published:** 2026-07-17

**Authors:** Yusuke Kakei, Keitaro Senoo, Arito Yukawa, Kohei Kawai, Masahiro Makino, Jun Munakata, Nobunari Tomura, Satoshi Shimoo, Hirokazu Shiraishi, Satoaki Matoba

**Affiliations:** 1 Department of Cardiovascular Medicine, Graduate School of Medical Science, Kyoto Prefectural University of Medicine, Kyoto, Japan; 2 Department of Cardiac Arrhythmia Research and Innovation, Graduate School of Medical Science, Kyoto Prefectural University of Medicine, Kyoto, Japan; Christus Oschner St. Patrick Hospital, UNITED STATES OF AMERICA

## Abstract

**Background:**

Home ECG can detect atrial fibrillation (AF), but patients’ hospital visit behavior after “possible AF” alerts remains uncertain. We assessed the rates, timing, and correlates of hospital visits following “possible AF” alerts in older adults with hypertension.

**Methods and findings:**

In a nationwide, decentralized cohort in Japan, 4,078 individuals aged ≥60 years enrolled; 3,820 were analyzed after exclusions. Participants used a home BP monitor equipped with a 30-s single-lead ECG capable of detecting “possible AF”, twice daily for 3 months. Of 1,700 participants who received ≥1 “possible AF” alert, 399 (23.5%) reported an alert-prompted healthcare facility visit within 3 months, and 289 (17.0%) underwent additional testing. Among 175 participants with valid visit dates, the median time to visit was 28 days (mean 36.4). In multivariable models, factors associated with lower odds of a hospital visit included age < 65 years (OR 0.75, 95% CI 0.59–0.95, p = 0.016), current drinking (OR 0.73, 0.57–0.95, p = 0.018), no daily palpitations (OR 0.69, 0.50–0.95, p = 0.022), and no palpitations on the alert day (OR 0.51, 0.30–0.85, p = 0.010). Notably, 105 of 220 (47.7%) participants with physician-adjudicated AF remained without a clinical diagnosis at 12 months.

**Conclusions:**

In this cohort of older, hypertensive individuals, follow-up rates after a “possible AF” alert were low; fewer than one in four individuals visited a healthcare facility, and fewer than one in five underwent additional testing within 3 months. Hospital visits were less common among younger participants, those who consume alcohol, and those without palpitations. These findings highlight significant gaps between mHealth alerts and subsequent clinical action.

## Introduction

The prevalence of atrial fibrillation (AF) and associated stroke is increasing with the progressive aging of the population. Early rhythm control strategies and catheter ablation have been reported as effective treatments for AF [1–3], highlighting the growing importance of early detection and intervention. In recent years, home-use devices equipped with AF detection capabilities, such as home electrocardiogram (ECG) monitors, have become widespread. One such example is the Complete (Omron Healthcare, Kyoto, Japan), a home blood pressure monitor with ECG functionality. It enables simultaneous blood pressure (BP) measurement and ECG recording and is utilized in various settings [4–6].

While home ECG monitoring devices equipped with algorithms capable of detecting rhythm irregularities suggestive of AF have become increasingly available, the subsequent rate of consultation with medical institutions following screening remains notably low. In fact, the AF-CATCH study, which used ECG screening, reported that among 103 individuals diagnosed with AF, only 17 consulted a specialist within one year [7]. Furthermore, follow-up rates in screening studies using photoplethysmography (PPG) were also modest: 57% in the Apple Heart Study, 35.3% in the Fitbit Heart Study, and 61.8% in the Huawei Heart Study. [8–10]

Thus, low follow-up rates after suspected AF detection appear to be a common issue, regardless of the screening modality (ECG or PPG). Recent large-scale randomized trials, including the VITAL-AF and GUARD-AF studies, have further demonstrated that while systematic screening can identify new AF cases, the clinical pathway from detection to treatment initiation remains challenging [11–13]. However, the factors that hinder patients from seeking medical consultation after screening are not well understood.

Therefore, this study investigated healthcare-seeking behaviors following AF screening as a sub-analysis of the Omron Heart Study, an AF screening trial targeting hypertensive patients aged 60 years or older. The Omron Heart Study was a prospective, multicenter observational study conducted across Japan from April 2022 to July 2023, which enrolled 4,078 hypertensive patients aged 60 years or older to evaluate home-based ECG and blood pressure monitoring for AF screening.

## Methods

To include a geographically and demographically diverse population, we enrolled participants using a decentralized clinical trial (DCT) approach. During the enrollment period, we invited users of the OMRON Connect smartphone application (Omron Healthcare Co., Ltd., Kyoto) via in-app notifications. OMRON Connect is a health-management app provided by Omron Healthcare that enables seamless transfer and management of data from Omron blood pressure monitors, body composition scales, and pedometers via Bluetooth. To avoid restricting recruitment to technology-savvy users, we additionally recruited volunteers aged ≥60 years who did not use OMRON Connect via a study website.

Participants reviewed study information on the website and were directed to an online application. Eligibility was screened online; those deemed eligible provided electronic informed consent on the website. The study office then mailed a Complete device to each enrolled participant. Participants recorded ECGs and blood pressure for 3 months after receipt of the device.

The protocol was approved by the Ethics Review Board of Kyoto Prefectural University of Medicine (approval No. ERB-C-1994). The dataset used for this secondary analysis was accessed for research purposes on 21 June 2024. During the data collection process, the authors had access to information that could identify individual participants, but only for research purposes and in accordance with ethical standards. Any information that could directly identify individual participants was not used in the analysis and was safeguarded to ensure confidentiality in line with privacy requirements.

### Eligibility

Inclusion criteria were: (1) a history of hypertension treated with antihypertensive medication; (2) age ≥ 60 years; and (3) provision of informed consent. Exclusion criteria were: (1) current participation in, or plans to participate in, an interventional trial; (2) self-reported prior physician-diagnosed AF; (3) self-reported use of oral anticoagulation; (4) implanted pacemaker and/or defibrillator; (5) residence outside Japan; and (6) any condition judged by the treating physician to make participation inappropriate.

### Device use and follow-up

Participants were instructed to obtain two measurements each morning and evening by touching the electrodes on the top and both sides of the Complete monitor, thereby recording a 30-second ECG concurrently with BP measurement, for a period of 3 months. All participants were instructed to sit with feet flat on the floor for 5 minutes before measurement and to allow a 30-second interval between the two measurements; the average of the two readings was used. During ECG recording with the Complete monitor, the OMRON connect smartphone application served as the display. When the algorithm detected a rhythm consistent with possible AF, the application showed a “possible AF” result. The alert indicated “possible atrial fibrillation” and advised the user to consult a physician. No active monitoring service or follow-up system was implemented to contact participants after receiving an alert. No specific reminder or follow-up was provided to participants who did not seek medical advice after receiving an alert, as the study was designed as an observational investigation of natural healthcare-seeking behavior.

A dedicated monitoring server tracked device usage and handled participant inquiries throughout follow-up. Each week, participants automatically received an email report from the study office summarizing their ECG measurement adherence for that week and the remaining days until study end. If adherence was 0% for three consecutive days, call-center staff telephoned the participant to check for device malfunction. At 3 months after enrollment, participants completed a questionnaire regarding care-seeking during the 3-month period; devices and questionnaires were returned to the study office. The 3-month questionnaire assessed whether participants had visited a healthcare facility in response to the device alert, whether additional testing had been recommended, whether they had received a new AF diagnosis, and whether anticoagulation therapy had been initiated. The questionnaire also inquired about palpitations both at the time of the alert and in daily life. The full questionnaire is provided as Supplementary Material (S1 Questionnaire). At 12 months after enrollment, participants completed a second questionnaire focusing on physician-diagnosed AF and subsequent clinical management.

### ECG adjudication

After the 3-month recording window, each 30-second ECG tracing was independently reviewed, under blinding, by six board-certified cardiologists. Tracings flagged as possible AF by the device were categorized as sinus rhythm, AF, or uninterpretable. For each tracing, three cardiologists were randomly assigned; AF was confirmed only when at least two of the three adjudications indicated AF. When the three readings were discordant, the tracing was reread and a consensus was reached through discussion. The ECG adjudication was performed after the 3-month recording period had ended, and the results were not communicated to participants. Therefore, participants’ healthcare-seeking behavior was based solely on the device alerts and their own initiative to seek care.

### Outcomes and statistical analysis

Baseline characteristics and questionnaire responses were summarized as counts (percentages) for categorical variables and mean (standard deviation) for continuous variables. Univariable and multivariable logistic regression analyses were used to examine factors associated with hospital visits. Patient-intrinsic baseline characteristics were analyzed using multivariable logistic regression, while device-extrinsic factors (e.g., alert frequency) were analyzed separately using univariable analyses to maintain clarity of interpretation. The time from the device AF alert to the first medical visit was modeled using Poisson regression to estimate incidence rate ratios (IRRs). Negative binomial regression was used when overdispersion was present. The Mann–Whitney U test was used as a non-parametric test to compare distributions between groups. A two-sided p-value < 0.05 was considered statistically significant. Analyses were performed using R (version 4.4.1), and results are presented as odds ratios (ORs) with 95% confidence intervals (CIs) and IRRs. Of the 399 participants who reported visiting a hospital within 3 months, 175 provided valid visit dates (i.e., dates within the 3-month observation period). The remaining 224 participants either did not provide a specific date or reported dates outside the observation window. The time-to-visit analysis (including Poisson/negative binomial regression and Mann–Whitney U tests) was limited to these 175 participants with valid visit dates.

## Results

### Study population

**Fig 1:**
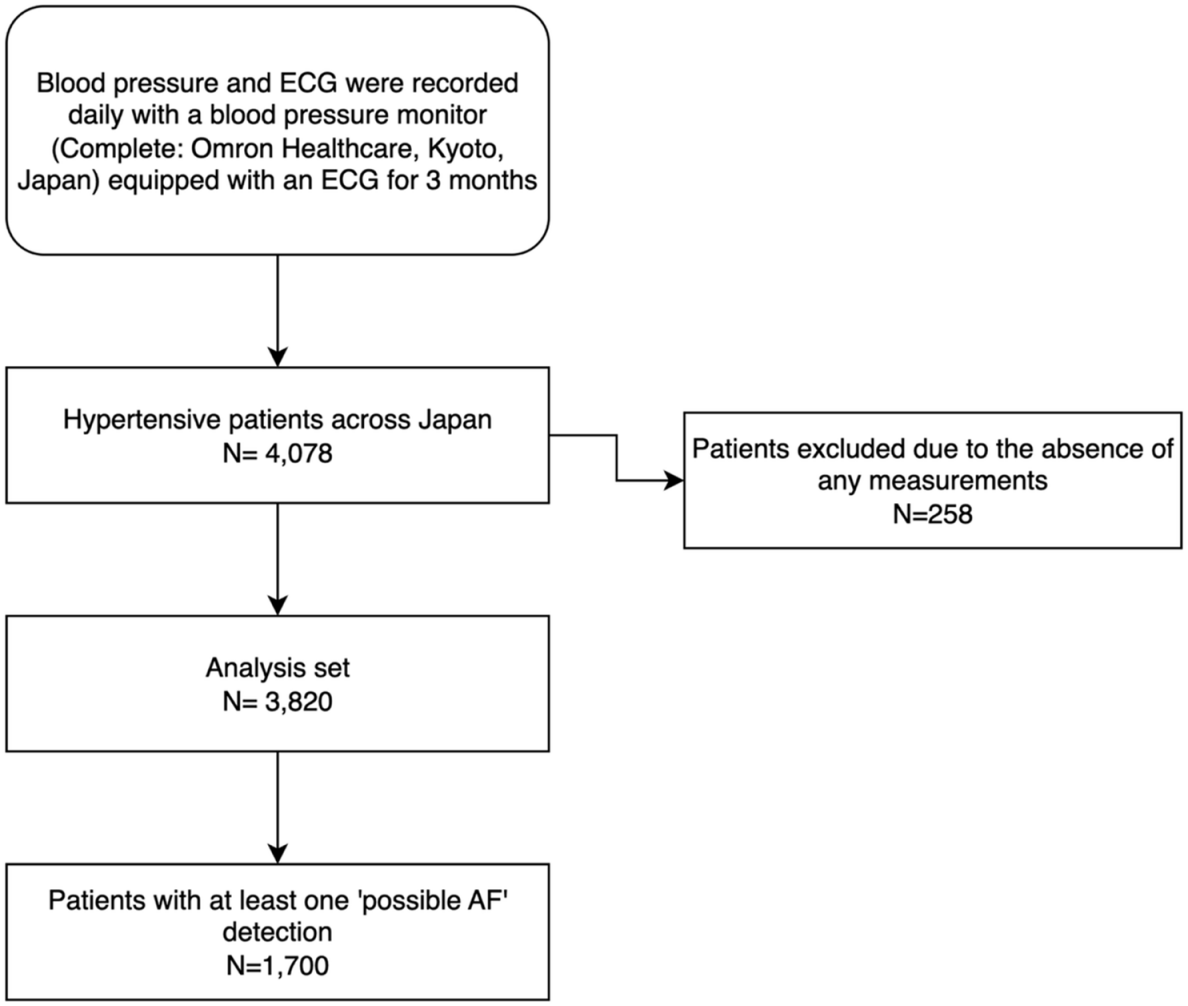
Schematic for study. Figure legend: Adults with hypertension aged ≥60 years were recruited nationwide (April 2022–July 2023). Participants used a home blood pressure monitor equipped with a single-lead electrocardiogram (ECG) (Complete; Omron Healthcare, Kyoto, Japan) to record blood pressure and a 30 s ECG twice daily for 3 months. Of 4,078 enrollees, 258 were excluded for absence of any measurements, leaving 3,820 in the analysis set. During the recording window, 1,700 participants received at least one device-generated “possible AF” notification. Abbreviations: AF, atrial fibrillation; ECG, electrocardiogram.

From April 2022 through July 2023, 4,078 individuals with hypertension across Japan enrolled. Of these, 2,182 (53.5%) were recruited through the OMRON connect smartphone application and 1,896 (46.5%) through a website. After excluding 258 for insufficient measurement data, 3,820 participants were analyzed. During the 3-month measurement period, 1,700 participants had at least one “possible AF” notification by the device (Fig 1). Physician adjudication of ECG waveforms confirmed AF in 220 participants. Of the 220 participants with physician-adjudicated AF, 218 (99.1%) responded to the 3-month follow-up questionnaire and 176 (80.0%) responded to the 12-month follow-up questionnaire. Baseline characteristics of the 1,700 participants with a possible AF notification are shown in Table 1.

**Table 1:** Baseline characteristics of participants with “possible AF”.

Variables	N = 1,700
Age, mean (SD)	66.8 (5.6)
60–64 (%)	705 (41.5%)
65–74 (%)	826 (48.6%)
≥ 75 (%)	169 (9.9%)
Height (cm), mean (SD)	166.0 (8.0)
Weight (kg), mean (SD)	66.6 (11.8)
BMI (kg/m²), mean (SD)	24.1 (3.3)
Sex (Male)	1325 (78.0%)
Smoking history	
Current	147 (8.6%)
Former	947 (55.7%)
Never	606 (35.6%)
Alcohol use	
None	488 (29.4%)
A few times a month	291 (17.5%)
2–3 times a week	287 (17.3%)
Daily	596 (35.9%)
Medical history	
Heart failure	25 (1.5%)
Diabetes mellitus	265 (15.6%)
Stroke/TIA	90 (5.3%)
Vascular disease	85 (5.0%)
Medications	
ACEi or ARB	1099 (65.3%)
CCB	1208 (71.8%)
Diuretic	138 (8.2%)
BB (including αβ-blocker)	96 (5.7%)
αβ-blocker	47 (2.8%)
α blocker	30 (1.8%)
ARNI	29 (1.7%)

Abbreviations: TIA, transient ischemic attack; ACEi, angiotensin-converting enzyme inhibitors; ARB, angiotensin II receptor blockers; ARNI, angiotensin receptor-neprilysin inhibitor; BB, beta blocker; CCB, calcium channel blocker.

Note: Antihypertensive drug use information was available for 1,682 patients. Data are presented as mean (standard deviation) for the continuous variables and number of subjects (%) for the categorical variables.

### Questionnaire responses at 3 and 12 months

Questionnaire results at 3 and 12 months are summarized in Table 2. At 3 months, we obtained questionnaire responses from 1,682 participants (98.9%); at 12 months, from 1,323 (77.8%). At 3 months, 399 participants (23.5%) reported visiting a healthcare facility in response to the device display. Additional testing had been performed in 289 (17.0%); 35 (2.1%) had been diagnosed with AF; and 18 (1.1%) had initiated anticoagulation. Between 3 and 12 months, an additional 80 participants (4.7%) reported a new AF diagnosis, 10 (0.6%) initiated anticoagulation, 2 (0.1%) underwent electrical cardioversion, and 14 (0.8%) underwent catheter ablation. Altogether, 115 participants had an AF diagnosis in clinical care within 12 months, corresponding to 52.3% of the 220 adjudicated as AF by physician review.

**Table 2:** 3-month and 12-month surveys of possible AF detected patients.

New self-reported events since enrolling in the study	3-Month survey after study enrollment (n = 1,682)	12-Month survey after study enrollment (n = 1,323)
Contact with a healthcare provider	399 (23.5%)	—
Recommend to have additional testing	289 (17%)	—
Recommended to see a specialist	45 (2.6%)	—
Received a new diagnosis of AF	35 (2.1%)	80 (4.7%)
Prescribed anticoagulants for AF	18 (1.1%)	10 (0.6%)
Underwent a cardioversion for AF	1 (0.1%)	2 (0.1%)
Underwent a catheter ablation for AF	3 (0.2%)	14 (0.8%)

Values in the 12-month column represent new events between 3 and 12 months, not cumulative events since enrollment.

### Factors associated with hospital visits

In multivariable logistic regression with the outcome of a medical visit within 3 months, age < 65 years, current alcohol use, absence of palpitations at the time of the device alert, and absence of palpitations in daily life were each associated with significantly lower odds of visiting a healthcare facility. Specifically, participants without palpitations on the alert day were approximately half as likely to visit a healthcare facility (OR 0.51), and those without daily palpitations were also less likely to seek care (OR 0.69). Participants aged <65 years were approximately 25% less likely to visit (OR 0.75), and current drinkers were approximately 27% less likely (OR 0.73). In the three-level smoking history comparison (current, former, none), using never-smoking as the reference, past smoking was significantly associated with visiting a healthcare facility (Table 3). We evaluated interactions among key variables; none were both statistically significant and clinically interpretable. Among the 175 participants with valid visit dates, the mean and median times from the first device alert to the medical visit were 36.4 and 28 days, respectively. No candidate covariate showed a significant association with time to visit.

**Table 3:** Logistic regression for factors associated with a hospital visit.

	Univariable analysis			Multivariable analysis		
Variable	OR	95%CI	P value	OR	95%CI	P value
Age (<65y/ ≥ 65y)	0.76	0.60–0.95	0.018	0.75	0.59–0.95	0.016
Sex (male/ female)	0.98	0.75–1.28	0.872	0.93	0.67–1.27	0.631
Smoking history (former/ never)	1.32	1.03–1.69	0.028	1.42	1.07–1.87	0.015
Smoking history (current/ former)	1.04	0.70–1.55	0.852	1.06	0.70–1.59	0.787
Smoking history (current/ never)	1.37	0.90–2.08	0.142	1.5	0.96–2.33	0.074
Alcohol (drinker/ non-drinker)	0.73	0.58–0.93	0.011	0.73	0.57–0.95	0.018
History of heart failure (present/ absent)	1.55	0.66–3.61	0.314	1.31	0.55–3.12	0.541
History of diabetes (present/ absent)	1.18	0.87–1.59	0.283	1.13	0.83–1.54	0.450
History of stroke/TIA (present/ absent)	0.99	0.60–1.64	0.975	0.94	0.57–1.57	0.825
History of vascular disease (present/ absent)	1.56	0.97–2.49	0.066	1.44	0.89–2.35	0.139
Daily palpitations (no/ yes)	0.6	0.44–0.80	<0.001	0.69	0.50–0.95	0.022
Palpitation on the AF day (no/ yes)	0.41	0.25–0.66	<0.001	0.51	0.30–0.85	0.010

Abbreviations: OR: odds ratio; CI: confidence interval; TIA: transient ischemic attack; AF: atrial fibrillation. For binary variables, presence of the condition is compared against its absence unless otherwise stated.

### Association between alert frequency and hospital visits

We also examined the relationship between the number of “possible AF” detections and hospital visit. A greater number of possible AF detections on the first alert day was associated with higher odds of visiting within 3 months (OR 1.25, 95% CI 1.09–1.42, p = 0.0009). In contrast, the association with time to visit was not significant (IRR 0.95, 95% CI 0.85–1.06, p = 0.34). When possible AF was displayed on 2 consecutive days, visiting within 3 months was more likely (OR 2.22, 95% CI 1.62–3.04, p < 0.001), whereas the association with time to visit was not significant (IRR 0.82, 95% CI 0.58–1.18, p = 0.29).

## Discussion

In our follow-up investigation, despite receiving a “possible AF” notification from the home device, only 399 patients (23.5%) visited a medical institution specifically in response to the alert within 3 months. Additional diagnostic testing was performed in only 289 patients (17.0%). The median time to consultation was approximately one month. Notably, the Omron Heart Study cohort, from which our sample was drawn, was recruited from “OMRON Connect” app users and volunteers, potentially representing a population with relatively high health consciousness. This selection bias suggests that the consultation rate in a real-world, general consumer setting might be even lower.

It is important to note that only 220 of 1,700 participants (12.9%) who received “possible AF” alerts were confirmed to have AF by physician adjudication, meaning that approximately 87% of alerts were not confirmed as AF. This high rate of unconfirmed alerts provides critical context for interpreting the observed low follow-up rate of 23.5%. The decision not to seek medical care after receiving an alert may, in part, represent a reasonable response, particularly among participants who received only a single alert. It should be emphasized that the behavioral analysis in this study examines responses to “possible AF” alerts from the device, not confirmed AF episodes. Future digital health interventions should consider strategies to improve the positive predictive value of alerts to enhance appropriate healthcare-seeking behavior.

The effectiveness of cardiovascular screening programs has long been challenged by low adherence to follow-up consultations [14,15]. AF screening faces a similar issue, with low rates of subsequent definitive diagnosis and initiation of anticoagulation therapy being reported [16]. In our prior study, the Omron Heart Study, the detection rate of undiagnosed AF in hypertensive patients aged 60 years or older was 6.38% (197/3087) in men and 3.15% (23/731) in women [17]. The low consultation rate observed in our study therefore represents a significant public health challenge, and identifying the barriers to post-screening medical consultation is crucial.

In our logistic regression analysis of factors related to hospital visit behavior, characteristics significantly associated with not visiting a medical institution included: age < 65 years, current alcohol consumption, and the absence of palpitations (both during the ECG recording and in daily life). In contrast, compared to the never-smoking group, the former-smoking group was significantly associated with a higher likelihood of visiting hospital. The tendency for lower follow-up rates among younger individuals has been reported previously in cardiovascular screening [15]. The low consultation rate observed in our “< 65 years” group may be partially attributable to difficulties in securing time for medical appointments, possibly due to employment status. It has been noted that AF patients often do not perceive their symptoms as severe, which can contribute to delays in visiting hospital [18,19]. Our finding that the absence of self-reported palpitations (either at the time of the device notification or in daily life) was significantly associated with non-consultation aligns with this previous research. Regarding alcohol habits, previous reports indicate that the frequency of outpatient visits and medical expenditures are inversely correlated with alcohol consumption [20–22], suggesting that individuals who consume alcohol may utilize healthcare services less frequently. The association we observed between “current alcohol consumption” and weaker hospital visit behavior is consistent with these findings. Potential mechanisms may include lower health literacy among heavy drinkers, leading to reduced consultation rates [23]. Additionally, alcohol consumption may serve as a proxy for unmeasured socioeconomic or psychosocial factors, including health anxiety levels or attitudes toward preventive care. Individuals who consume alcohol regularly may have competing priorities that reduce engagement with the healthcare system. However, these associations should be interpreted with caution given the lack of data on socioeconomic status and health literacy in our study.

Furthermore, our study highlighted challenges in the medical response following consultation. At 3 months, among the 399 patients who sought care for a “possible AF” alert, only 289 (72.4% of those visiting) underwent additional diagnostic testing. At that time, 35 patients had been diagnosed with AF, and anticoagulation therapy had been initiated in 18. Over the cumulative 12-month period, 115 patients were diagnosed with AF and 28 initiated anticoagulation therapy. This variability in post-consultation evaluation and treatment initiation may stem from provider-side concerns about the accuracy and reliability of digital devices [24], as well as documented knowledge and skill gaps in AF care among different medical specialties and professions [25–28].

Based on these findings, the factors hindering appropriate medical follow-up after a “possible AF” alert can be broadly categorized into two groups. The first group comprises patient-side barriers to hospital visit, including constraints on opportunities (e.g., age, employment) and a lack of perceived clinical significance (e.g., absence of symptoms, alcohol use). The second group involves provider-side variability in response, potentially driven by concerns about device reliability or gaps in AF care knowledge.

Perhaps the most consequential finding of this study is that 105 of 220 (47.7%) participants with physician-adjudicated AF remained without a clinical AF diagnosis at 12 months. This highlights a critical gap in the pathway from home-based screening to clinical diagnosis and treatment. Given the well-established benefits of anticoagulation therapy for stroke prevention in AF, the failure to achieve clinical diagnosis in nearly half of confirmed AF cases represents a significant missed opportunity for intervention. These findings underscore the need for improved systems to facilitate timely clinical evaluation following positive screening results from home monitoring devices.

Given the current landscape where the proliferation of digital devices may increase the detection of subclinical (“silent”) AF, establishing a framework to connect suspected cases to definitive diagnosis and treatment initiation is urgently needed. To achieve this, strengthening patient-centered health literacy support is likely beneficial. This support should move beyond one-size-fits-all education and include standardized information delivered at timely intervals, coupled with navigation assistance (e.g., guidance on where to seek care, consultation resources) tailored to user characteristics. Concurrently, our findings suggest a strong need for fostering a shared understanding of the clinical significance and limitations of these digital devices among both patients and providers. Furthermore, enhancing the infrastructure to support appropriate evaluation and response in clinical settings—through guideline dissemination and implementation support—is warranted [29–33].

### Limitations

This study has several limitations. First, response rates were high at 3 months (98.9%) but lower at 12 months (77.8%), which may have led to under-ascertainment of actual hospital visit and AF diagnoses. Second, of the 399 who visited care after an alert, only 175 provided valid visit dates within the observation period; the time-to-visit analysis was limited to these participants. Third, follow-up relied on self-report and is therefore susceptible to recall and misclassification bias for AF diagnoses and anticoagulation initiation. Fourth, generalizability is limited: participants were aged ≥60 years with hypertension in Japan, and results may differ in other age groups or clinical backgrounds. Fifth, hospital visits were self-reported and were not validated against medical records, which may introduce recall bias or social desirability bias. Sixth, although the device algorithm has been validated in prior studies, we do not have device-specific accuracy data (sensitivity, specificity, or false-positive rate) for this particular population of older hypertensive adults. Seventh, the study did not collect data on reasons why participants chose not to seek medical care after receiving an alert, limiting our ability to distinguish between intentional non-response and barriers to access. Eighth, healthcare system factors such as appointment availability, geographic access, and cost barriers were not assessed and may have influenced follow-up rates. Ninth, variables such as urban versus rural residence, access to healthcare facilities, and prior healthcare utilization patterns were not available in this study.

## Conclusion

In this study, despite receiving a “possible AF” notification from a home ECG monitor, only 23.5% of patients visited a medical institution in response to the alert, and only 17.0% underwent additional testing. The time interval from notification to visit was approximately one month. Logistic regression analysis revealed that age < 65 years, current alcohol use, and the absence of palpitations (both at the time of the alert and in daily life) were associated with not visiting hospital after the notification. However, the low follow-up rate should be interpreted in the context that approximately 87% of “possible AF” alerts were not confirmed as AF, and non-response may partly reflect reasonable decision-making. Furthermore, 105 of 220 (47.7%) participants with physician-adjudicated AF remained without a clinical AF diagnosis at 12 months, highlighting a critical gap in the pathway from screening to treatment.

## Supporting information

S1 Questionnaire Three-month follow-up questionnaire administered to study participants regarding healthcare-seeking behavior after a “possible AF” alert. (DOCX)
