# Supplementary material for: Rates and predictors of hospital visits after “Possible AF” alerts from a home ECG monitor in older adults with hypertension: A sub-analysis of the Omron Heart Study
Source: PLoS One. 2026 Jul 17;21(7):e0353867. doi: 10.1371/journal.pone.0353867 (PMC13379017; doi:10.1371/journal.pone.0353867)
Supplement: S1 Questionnaire — (DOCX) [file pone.0353867.s001.docx]

**S1 Questionnaire**

*English Translation of Study Questionnaires*

# **Part A: Baseline Questionnaire (Pre-Study)**

*(To be completed before the start of the 3-month measurement period)*

Thank you for participating in the Atrial Fibrillation Screening Study in Patients with Hypertension.

Please complete this questionnaire before the study begins.

** We may contact you by telephone at a later date to confirm the content of this questionnaire.*

**Q1.** Are you currently taking medication to lower your blood pressure? (Circle ① Yes or ② No)

① Yes (Please list the medication names below, to the extent you know them)

1: ____________________

2: ____________________

3: ____________________

4: ____________________

② No

**Q2.** Do you smoke? (Circle ① Yes or ② No)

① Yes (Please provide your smoking details below)

How many cigarettes do you smoke per day? ________ cigarettes

How many years have you been smoking? ________ years

② No

**Q3.** Do you drink alcohol? (Circle the applicable number)

① Every day ② 2–3 times per week ③ A few times per month ④ Never

**Q4.** What is your waist circumference? ________ cm

**Q5.** Do you have any subjective symptoms? (Circle all applicable numbers)

① Palpitations ② Shortness of breath ③ Dyspnea ④ Dizziness ⑤ Chest pain ⑥ Chest discomfort

If none of the above apply, please describe freely:

( ______________________________________________________________ )

**This is the end of the pre-study questionnaire. Thank you for your time.**

# **Part B: 3-Month Follow-Up Questionnaire**

*(To be completed after the end of the 3-month measurement period)*

Thank you for continuing the measurements for 3 months. Please complete this final questionnaire.

** We may contact you by telephone at a later date to confirm the content of this questionnaire.*

**Q1.** During the past 3 months, did you visit a healthcare facility (e.g., your primary care physician) based on the results displayed by the electrocardiogram device? (Circle ① Yes or ② No)

① Yes

② No

*If you answered “No” to Q1, the questionnaire is complete. The following questions are for those who answered “Yes” only.*

**Q2.** Before visiting the healthcare facility, did you use a remote health consultation service to receive advice from a physician regarding the results? (Circle ① Yes or ② No)

① Yes

② No

**Q3.** How many times did you visit a healthcare facility? (Circle the applicable number)

① 1 time ② 2 times ③ 3 times ④ 4 times ⑤ 5 or more times

(Free entry: ________ times)

**Q4.** Please provide the date of your first visit (in the Western calendar).

Date (YYYY / MM / DD): ____________________

**Q5.** Were any tests performed during your visit? (Circle ① Yes or ② No)

① Yes (Circle all applicable tests below)

a. 12-lead electrocardiogram b. 24-hour Holter electrocardiogram c. Blood tests

d. Chest X-ray e. Chest CT scan f. MRI

② No

**Q6.** During the past 3 months, were you referred to a specialist by your primary care physician? (Circle ① Yes or ② No)

① Yes

② No

**Q7.** During the past 3 months, did you receive any hospitalization or emergency treatment? (Circle ① Yes or ② No)

① Yes (Circle all applicable treatments below)

a. Medication administration (Drug name: __________________ )

b. Electrical cardioversion (also known as electrical defibrillation)

c. Catheter ablation for atrial fibrillation

d. Pacemaker implantation

e. Other (please specify: __________________ )

② No

**Q8.** During the past 3 months, have you been newly diagnosed with any of the following conditions? (Circle all applicable conditions)

a. Atrial fibrillation b. Atrial premature contractions c. Ventricular premature contractions

d. Cerebral infarction / Transient ischemic attack e. Heart failure

f. Myocardial infarction / Angina pectoris g. Mitral stenosis h. Mitral regurgitation

i. Aortic stenosis j. Aortic regurgitation

*The following questions (Q9–Q12) are for those who answered “Atrial fibrillation” in Q8 only.*

**Q9.** Were you newly prescribed any medication for atrial fibrillation? (Circle ① Yes or ② No)

① Yes

② No

*The following questions (Q10–Q12) are for those who answered “Yes” to Q9 only.*

**Q10.** Which of the following medications were prescribed? (Circle all applicable numbers)

① Lixiana (edoxaban) ② Eliquis (apixaban) ③ Pradaxa (dabigatran)

④ Xarelto (rivaroxaban) ⑤ Warfarin

**Q11.** When was the medication started?

Date (YYYY / MM / DD): ____________________

**Q12.** Have you experienced any bleeding since starting the medication? (Circle ① Yes or ② No)

① Yes (Circle the applicable site of bleeding)

a. Subcutaneous hemorrhage b. Gastrointestinal bleeding (e.g., gastric, rectal, hemorrhoidal)

c. Intracranial hemorrhage

② No

**This is the end of the 3-month follow-up questionnaire. Thank you for your time.**

# **Part C: 9-Month Post-Measurement Follow-Up Questionnaire**

*(Administered 9 months after the end of the 3-month measurement period, i.e., 12 months from study enrollment)*

This survey targets participants who received a **"possible atrial fibrillation"** alert at least once during the 3-month measurement period. If this questionnaire was not returned, a follow-up survey may be conducted at a later date.

*Note: No compensation (e.g., QUO card) will be provided for completing this questionnaire.*

**Q1.** Among the following medications, are you still currently taking any that were started during the 3-month measurement period?

Medications: Lixiana (edoxaban), Eliquis (apixaban), Pradaxa (dabigatran), Xarelto (rivaroxaban), Warfarin

① Yes (Circle all applicable medications)

① Lixiana ② Eliquis ③ Pradaxa ④ Xarelto ⑤ Warfarin

② No

**Q2.** Between the end of the measurement period and now, have you received a new diagnosis of atrial fibrillation at a hospital?

① Yes

② No

**Q3.** Among the following medications, have any been newly prescribed (or switched to) for atrial fibrillation after the 3-month measurement period?

Medications: Lixiana (edoxaban), Eliquis (apixaban), Pradaxa (dabigatran), Xarelto (rivaroxaban), Warfarin

① Yes (Circle all applicable medications)

① Lixiana ② Eliquis ③ Pradaxa ④ Xarelto ⑤ Warfarin

② No

*Q4 and Q5 are for those who answered “Yes” to Q3 only.*

**Q4.** When was the medication started?

Date (YYYY / MM / DD): ____________________

**Q5.** Have you experienced any bleeding since starting the medication?

① Yes (Circle the applicable site of bleeding)

a. Subcutaneous hemorrhage b. Gastrointestinal bleeding (e.g., gastric, rectal, hemorrhoidal) c. Intracranial hemorrhage

② No

**Q6.** Between the end of the measurement period and now, did you receive any hospitalization or emergency treatment?

① Yes (Circle all applicable treatments)

a. Medication administration (Drug name: __________________ )

b. Electrical cardioversion (also known as electrical defibrillation)

c. Catheter ablation for atrial fibrillation

d. Pacemaker implantation

e. Other (please specify: __________________ )

② No

**Q7.** Between the end of the measurement period and now, has the frequency of your visits to a healthcare facility (e.g., primary care physician) changed compared to during the 3-month measurement period?

① Yes, it changed. (Circle the applicable number)

1. Decreased 2. Increased

② No, it has not changed.

**Q8.** Between the end of the measurement period and now, have you been newly diagnosed with any disease?

① Yes (Please provide the disease name)

( ________________ ) ( ________________ ) ( ________________ )

② No

**This is the end of the questionnaire. Thank you very much for your participation.**
