# Supplementary material for: Rates and predictors of hospital visits after “Possible AF” alerts from a home ECG monitor in older adults with hypertension: A sub-analysis of the Omron Heart Study
Source: PLoS One. 2026 Jul 17;21(7):e0353867. doi: 10.1371/journal.pone.0353867 (PMC13379017; doi:10.1371/journal.pone.0353867)
Supplement: Attachment — Submitted filename: Response_to_Reviewers_PONE-D-25-63371_v2.docx [file pone.0353867.s003.docx]

**Kyoto Prefectural University of Medicine**

465 Kajii-cho, Kamigyo-ku, Kyoto 602-8566, JAPAN

Phone.+81-75-251-5111 Fax.+81-75-211-7093

Dear Academic Editor and Reviewers,

We thank you all for reading and commenting on the manuscript entitled *"Rates and Predictors of Hospital Visits After 'Possible AF' Alerts from a Home ECG Monitor in Older Adults with Hypertension: A Sub-analysis of the Omron Heart Study"* (PONE-D-25-63371). We appreciate your constructive feedback and have carefully addressed each point raised during the review process. Below, we provide a point-by-point response to the editor's and reviewers' comments. Changes to the manuscript are indicated in blue text.

We hope you will consider the revised version of the manuscript suitable for publication.

We are looking forward to hearing from you.

On behalf of the authors,

Yusuke Kakei, MD

**Comments from the Academic Editor**

**Comment 1**

*Only 220 of the 1,700 alerted participants had physician-adjudicated AF, meaning ~87% of "possible AF" alerts were not confirmed. Please integrate this context into the discussion and conclusions rather than attributing low follow-up rates primarily to patient-side barriers.*

**>> Thank you for this important comment.**

We fully agree that the high rate of unconfirmed alerts (~87%) is critical context for interpreting the low follow-up rate. In the revised manuscript, we have substantially expanded the Discussion to address this point. Specifically, we now discuss that the low follow-up rate may partly reflect a rational response to the high false-positive rate of "possible AF" alerts, rather than solely reflecting patient-side barriers such as lack of awareness or motivation.

The following text has been added to the Discussion:

It is important to note that only 220 of 1,700 participants (12.9%) who received "possible AF" alerts were confirmed to have AF by physician adjudication, meaning that approximately 87% of alerts were not confirmed as AF. This high rate of unconfirmed alerts provides critical context for interpreting the observed low follow-up rate of 23.5%. The decision not to seek medical care after receiving an alert may, in part, represent a reasonable response, particularly among participants who received only a single alert. Future digital health interventions should consider strategies to improve the positive predictive value of alerts to enhance appropriate healthcare-seeking behavior.

**Comment 2**

*The 12-month column of Table 2 requires clarification as to whether reported events are cumulative since enrollment or incident between months 3-12; in particular the identical anticoagulation figures at both timepoints (18, 1.1%) despite additional AF diagnosis by 12 months requires explicit explanation.*

**>> Thank you for pointing out this important issue.**

The 12-month column in Table 2 represents new events between 3 and 12 months, not cumulative values. We acknowledge that the identical anticoagulation figures at both timepoints was an error in our original manuscript. Upon re-examination of our data, 18 participants initiated anticoagulation at 3 months, and an additional 10 participants were newly prescribed anticoagulation between 3 and 12 months. The 12-month column now correctly shows 10 (0.6%) as new events.

We have corrected Table 2 accordingly and added a footnote clarifying that the 12-month column shows new events between 3 and 12 months. We also corrected the catheter ablation figures: 3 participants (0.2%) at 3 months and 14 participants (0.8%) as new events at 12 months.

**Comment 3**

*The finding that 105 of 220 cardiologist-adjudicated AF cases received no clinical diagnosis within 12 months is perhaps the most consequential observation in the manuscript and deserves prominent discussion as a public health implication rather than a passing mention.*

**>> Thank you for highlighting this critical point.**

We agree that this finding carries significant public health implications. In the revised manuscript, we have expanded the Discussion to give this observation more prominence. We now discuss the gap between device-detected AF and clinical diagnosis as a systemic issue that may reflect challenges in the current healthcare pathway from screening alerts to definitive diagnosis. Of the 220 participants with physician-adjudicated AF, 218 responded to the 3-month survey and 176 responded to the 12-month survey. Notably, the ECG adjudication results were not communicated to participants, meaning that these 105 undiagnosed individuals were unaware of their AF status throughout the study period.

The following text has been added to the Discussion:

Perhaps the most consequential finding of this study is that 105 of 220 (47.7%) participants with physician-adjudicated AF remained without a clinical AF diagnosis at 12 months. This highlights a critical gap in the pathway from home-based screening to clinical diagnosis and treatment. Given the well-established benefits of anticoagulation therapy for stroke prevention in AF, the failure to achieve clinical diagnosis in nearly half of confirmed AF cases represents a significant missed opportunity for intervention. These findings underscore the need for improved systems to facilitate timely clinical evaluation following positive screening results from home monitoring devices.

**Reviewer #1**

We thank Reviewer #1 for the thorough and constructive review. Here is a point-by-point response to each comment.

**Introduction**

*It flows well and references relevant literature. There are however additional references that could be included here or in the discussion, such as the VITAL-AF (2024), GUARD-AF (2024), and JAMA Cardiol 2021;6;(5):558-567.*

**>> Thank you for the suggestion. We have added references to the VITAL-AF, GUARD-AF, and the cited JAMA Cardiology article in the Introduction section where relevant.**

**Methods Q1: Alert content and monitoring service**

*The authors need to describe the alerts shared with the subjects. Was there a role for the monitoring service? Did they receive in addition to the notification something that advises them to seek medical care? It is important to clarify these points.*

**>> Thank you for this important question.**

Participants used the Complete (Omron Healthcare, Kyoto, Japan), a home blood pressure monitor equipped with single-lead ECG functionality. During ECG recording, the OMRON connect smartphone application served as the display, showing the analysis result including "possible AF" when detected. The alert indicated "possible atrial fibrillation" and advised the user to consult a physician. There was no active monitoring service or follow-up system that contacted participants after an alert.

We have added the following clarification to the Methods:

During ECG recording with the Complete monitor, the OMRON connect smartphone application served as the display. When the algorithm detected a rhythm consistent with possible AF, the application showed a "possible AF" result. The alert indicated "possible atrial fibrillation" and advised the user to consult a physician. No active monitoring service or follow-up system was implemented to contact participants after receiving an alert.

**Methods Q2: Reminders and follow-up**

*What happened to subjects if they did not seek medical advice after an alert? Was there any specific reminder follow-up?*

**>> No specific reminder or follow-up was provided to participants who did not seek medical advice after receiving an alert. The study was designed as an observational investigation of natural healthcare-seeking behavior following alerts, and therefore no intervention was implemented to encourage follow-up visits. We have clarified this in the revised Methods section.**

**Methods Q3: Notification to AF-confirmed participants**

*What happened with the 220 diagnosed with AF by physician confirmation? Were the subjects notified?*

**>> The ECG adjudication by three blinded cardiologists was performed after the 3-month measurement period had ended, and the results were not communicated to participants. Therefore, the 220 participants confirmed to have AF were not notified of their diagnosis through the study. Their healthcare-seeking behavior and subsequent clinical diagnoses were based solely on the device alerts and their own initiative to seek care. We have clarified this point in the revised manuscript.**

**Methods Q4: Missing visit dates and Poisson model**

*The analysis of time to seeking medical care is problematic given that some did not have accurate visit times. The authors should state explicitly whether dates of visits were missing for some or many participants (indicate the number), and how this impacts the Poisson model.*

**>> Thank you for this important methodological point.**

Of the 399 participants who reported visiting a hospital within 3 months, 175 provided valid visit dates (i.e., dates within the 3-month observation period). The remaining 224 participants either did not provide a specific date or reported dates outside the observation window. The time-to-visit analysis (including Poisson/negative binomial regression for IRR estimation and Mann-Whitney U tests) was limited to the 175 participants with valid visit dates.

We acknowledge that this reduced sample may introduce selection bias if participants with missing dates differ systematically from those with valid dates. However, among the 175 participants analyzed, no candidate covariate (age, sex, smoking, alcohol, comorbidities, palpitations, AF alert frequency, or consecutive-day alerts) showed a significant association with time to visit (all Mann-Whitney P > 0.05; Poisson/negative binomial regression P > 0.05).

We have added this information explicitly to the Methods and Results sections of the revised manuscript.

**Methods Q5: Univariable analysis and variable selection**

*What were the results of the univariable analysis and what determined the inclusion of variables in the multivariable model? There are other variables that could be considered such as urban vs. rural residence, access to healthcare facilities, prior healthcare utilization patterns, etc.*

**>> Thank you for this suggestion.**

For the multivariable logistic regression model (Table 3), candidate variables were selected based on clinical relevance and prior literature. Patient-intrinsic factors (age, sex, smoking status, alcohol consumption, history of heart failure, diabetes, stroke/TIA, vascular disease, and palpitations) were included in the multivariable model as these represent established or plausible predictors of healthcare-seeking behavior.

We acknowledge that variables such as urban vs. rural residence, access to healthcare facilities, and prior healthcare utilization patterns may also influence follow-up behavior. Unfortunately, these data were not collected in the original study. We have added this as a limitation in the revised manuscript.

The results of both univariable and multivariable analyses for all candidate variables are presented in Table 3 of the manuscript.

**Methods Q6: Alert variables analyzed separately**

*Why were the number of alerts and the display on 2 consecutive days not included in the multivariable model, but analyzed separately?*

**>> Thank you for this methodologically important question.**

We made a deliberate analytical decision to separate patient-intrinsic factors (demographic and clinical characteristics such as age, sex, comorbidities, and symptoms) from device-extrinsic factors (alert frequency and consecutive-day alert patterns). Patient-intrinsic factors reflect baseline characteristics that exist independently of the screening device, whereas device-extrinsic factors are properties of the alert exposure itself.

This separation is a standard analytical approach in epidemiology and clinical research. Combining intrinsic patient characteristics with extrinsic exposure variables in a single model can complicate interpretation, as the effect of an exposure (e.g., alert frequency) adjusted for baseline characteristics has a different meaning than the effect of a baseline characteristic adjusted for exposure. We therefore analyzed patient-intrinsic factors in a multivariable model (Table 3) and device-extrinsic factors in separate univariable analyses to maintain clarity of interpretation.

In the revised manuscript, we have clarified this rationale in the Methods section and reported univariable odds ratios for the device-extrinsic factors: the number of "possible AF" alerts on the first day was significantly associated with hospital visits (OR 1.25, 95% CI 1.09–1.42, p = 0.0009), and alerts on two consecutive days were also significantly associated (OR 2.22, 95% CI 1.62–3.04, p < 0.001).

**Methods Q7: Questionnaire as supplementary material**

*It helps to give the questionnaire as supplementary material or else to describe a bit the types of questions asked.*

**>> Thank you for this suggestion. We have added a description of the key questionnaire items in the Methods section and included the full questionnaire as Supplementary Material (S1 Questionnaire).**

**Results Q8: Subheadings**

*Better to provide subheadings for the results section.*

**>> Thank you for this suggestion. We have added subheadings to the Results section to improve readability.**

**Results Q9: App users vs. website volunteers**

*The manuscript doesn't quantify what proportion came from app users vs. website volunteers.*

**>> Thank you for pointing this out.**

Of the 4,078 enrolled participants, 2,182 (53.5%) were recruited through the OMRON connect smartphone application and 1,896 (46.5%) through a website. We have added this information to the Results section.

**Results Q10: Additional 80 AF diagnoses and anticoagulation**

*There were additional 80 subjects who were diagnosed with AF. How were they discovered to have AF? It is unclear how many received anti-coagulation.*

**>> Thank you for this question.**

The additional AF diagnoses at 12 months were identified through the 12-month follow-up questionnaire, in which participants were asked whether they had received a new AF diagnosis since the 3-month survey. These diagnoses were made through routine clinical care (i.e., participants sought medical attention and were diagnosed by their physicians), not through the study's ECG adjudication process.

Regarding anticoagulation: as noted in our response to Editor Comment 2, the original Table 2 contained an error. The corrected figures show that 18 participants initiated anticoagulation at 3 months, and an additional 10 were newly prescribed between 3 and 12 months. We also corrected the catheter ablation figures: 3 at 3 months and 14 new cases at 12 months. The 12-month column now shows new events only. These have been corrected in the revised Table 2.

**Results Q11: Table 2 clarification**

*Table 2 is confusing: The 12-month column needed clarification regarding whether values represent cumulative or new events.*

**>> We agree that the original Table 2 was confusing. In the revised manuscript, we have added a footnote to Table 2 explicitly stating that the 12-month column represents new events between 3 and 12 months. We have also corrected the anticoagulation and catheter ablation figures as described above.**

**Results Q12: Clinical effect size description**

*When describing predictors, clarifying the clinical effect size would improve readability (e.g., "participants without palpitations were ~50% less likely to follow up").*

**>> Thank you for this helpful suggestion. We have revised the Results text to include clinically meaningful descriptions of effect sizes alongside the statistical values. For example, we now state that participants without palpitations on the alert day were approximately half as likely to seek medical care (OR 0.51).**

**Results Q13: 105 undiagnosed AF cases**

*Of 220 with confirmed AF, only 115 (52.3%) were diagnosed clinically within 12 months; what happened to the other 105?*

**>> This is addressed in our response to Editor Comment 3 above.**

To summarize: the ECG adjudication results were not communicated to participants, so the 105 participants with unconfirmed AF remained unaware of their AF status. They did not receive clinical AF diagnoses through routine care within the 12-month follow-up period. Of the 220 AF-confirmed participants, 218 responded to the 3-month survey and 176 responded to the 12-month survey, so the finding is not attributable to loss to follow-up. This observation has been given prominent discussion in the revised manuscript as a public health concern.

**Discussion Q14: False positive distinction**

*The paper should clarify how often alerts were false positives, whether false-positive alerts contributed to avoidance or skepticism, and that behavioral analysis is on alerts, not confirmed AF episodes.*

**>> Thank you for this critical comment. We have addressed this in the revised Discussion as described in our response to Editor Comment 1. We now explicitly state that the behavioral analysis examines responses to "possible AF" alerts (not confirmed AF episodes), and have also added discussion of the distinction between device-generated alerts and physician-confirmed AF.**

**Discussion Q15: 87% false-positive rate**

*Only 12.9% of "possible AF" alerts were confirmed. This 87% false-positive rate is a critical context for understanding low follow-up but is barely discussed.*

**>> We fully agree. This has been addressed comprehensively in the revised Discussion as detailed in our response to Editor Comment 1.**

**Discussion Q16: Alcohol and healthcare utilization**

*The inverse association between alcohol consumption and healthcare utilization is interesting but deserves deeper consideration. Is this confounded by unmeasured socioeconomic factors? Could alcohol consumption be a marker for lower health anxiety?*

**>> Thank you for this thoughtful comment.**

We agree that the inverse association between alcohol consumption and healthcare-seeking behavior warrants deeper consideration. In the revised Discussion, we have expanded this paragraph to acknowledge potential confounders. Alcohol consumption may serve as a proxy for unmeasured socioeconomic or psychosocial factors, including health literacy, health anxiety levels, or attitudes toward preventive care. Additionally, individuals who consume alcohol regularly may be less health-conscious overall or may have competing priorities that reduce engagement with the healthcare system. We have noted these possibilities in the revised manuscript and acknowledged the limitation of not having data on socioeconomic status and health literacy.

**Discussion Q17: Additional limitations**

*Limitations section misses important considerations: (a) No validation of self-reported hospital visits against medical records; (b) No information on device accuracy/false-positive rate in this specific population; (c) No data on reasons participants chose not to seek care; (d) No assessment of healthcare system factors (appointment availability, cost barriers).*

**>> Thank you for identifying these important limitations. We have added all four points to the Limitations section of the revised manuscript:**

Several additional limitations should be acknowledged. First, hospital visits were self-reported and were not validated against medical records, which may introduce recall bias or social desirability bias. Second, although the device algorithm has been validated in prior studies, we do not have device-specific accuracy data (sensitivity, specificity, or false-positive rate) for this particular population of older hypertensive adults. Third, the study did not collect data on reasons why participants chose not to seek medical care after receiving an alert, limiting our ability to distinguish between intentional non-response and barriers to access. Fourth, healthcare system factors such as appointment availability, geographic access, and cost barriers were not assessed and may have influenced follow-up rates. Fifth, variables such as urban versus rural residence, access to healthcare facilities, and prior healthcare utilization patterns were not available in this study.

**Reviewer #2**

*The Manuscript titled Rates and Predictors of Hospital Visits After "Possible AF" Alerts from a Home ECG Monitor in Older Adults with Hypertension: A Sub-analysis of the Omron Heart Study; is technically sound and the data support the conclusion. The manuscript is written in standard English and feels smooth while going through.*

**>> Thank you for your positive evaluation of our manuscript. We appreciate your careful review and encouraging comments.**

**Reviewer #3**

We thank Reviewer #3 for the constructive and insightful comments. Here is a point-by-point response.

**Line 46: Device accuracy statement**

*The authors state that these home devices have high accuracy in AF detection. What is the basis for this statement? In this study, only a minority of device-triggered alerts were ultimately adjudicated as AF, highlighting that alerts represent possible rather than confirmed AF. The wording should be adjusted to reflect this distinction.*

**>> Thank you for this important distinction.**

We agree that the original wording was misleading. The device is designed to detect rhythm irregularities suggestive of AF, not to provide a definitive diagnosis. The term "high accuracy" was based on prior validation studies of the device algorithm; however, in the context of our study where only 12.9% of alerts were confirmed as AF, this wording is inappropriate. We have revised the text to:

Home ECG monitoring devices equipped with algorithms capable of detecting rhythm irregularities suggestive of atrial fibrillation have become increasingly available.

**Methods: Omron Heart Study context**

*The authors should briefly describe the larger Omron heart study to provide some context.*

**>> Thank you for this suggestion. We have added a brief description of the parent Omron Heart Study in the Introduction:**

The Omron Heart Study was a prospective, multicenter observational study conducted across Japan from April 2022 to July 2023, which enrolled 4,078 hypertensive patients aged 60 years or older to evaluate home-based ECG and blood pressure monitoring for AF screening. Participants were recruited through the OMRON connect smartphone application (n = 2,182; 53.5%) and a website (n = 1,896; 46.5%). After excluding 258 participants for insufficient measurement data, 3,820 participants were included in the main analysis.

**Methods: ECG recording instructions**

*It would be helpful to explicitly state that participants were instructed to record ECGs twice daily for 3 months, as this is not immediately clear from the current description.*

**>> Thank you for this suggestion. The twice-daily ECG and blood pressure measurement protocol was described in the “Device use and follow-up” subsection of the Methods; however, we recognize that this information could be made more immediately accessible. We have revised the description to more clearly state the measurement frequency and the 3-month recording period.**

Participants were instructed to obtain two measurements each morning and evening by touching the electrodes on the top and both sides of the Complete monitor, thereby recording a 30-second ECG concurrently with BP measurement, for a period of 3 months.

**Results: AF alert denominator**

*Among the 1,682 respondents, how many had at least one AF alert? This would help contextualize the denominator for subsequent analyses.*

**>> Of the 3,820 participants in the main study, 1,700 received at least one "possible AF" alert during the 3-month monitoring period. Among these 1,700, 1,682 responded to the 3-month follow-up questionnaire (response rate: 98.9%). We have clarified this in the revised Results section.**

**Results: AF-confirmed questionnaire response**

*Among the 220 participants with physician-adjudicated AF, how many responded to the follow-up questionnaire?*

**>> Of the 220 participants with physician-adjudicated AF, 218 (99.1%) responded to the 3-month follow-up questionnaire and 176 (80.0%) responded to the 12-month follow-up questionnaire. We have added this information to the Results section.**

**Discussion: Nationwide estimates**

*The extrapolation to nationwide estimates of undiagnosed AF is an overreach. These estimates are based on a relatively small subgroup (220 adjudicated AF cases, with only 35 new diagnoses) and are not directly aligned with the primary objective of the study.*

**>> We agree with this assessment. The nationwide extrapolation was speculative and not well-supported by our data. We have removed this section from the revised manuscript.**

**Discussion: Neutral framing of follow-up rates**

*The interpretation of 'low follow-up rates' appears to be somewhat one-sided. A 'possible AF' alert from a home device does not represent a confirmed diagnosis. In many cases, deferring immediate medical evaluation may be reasonable.*

**>> We agree that the original framing was one-sided. As detailed in our response to Editor Comment 1, we have substantially revised the Discussion to present a more balanced interpretation, acknowledging that non-follow-up may partly reflect reasonable decision-making in the context of a high false-positive alert rate (~87%).**

**Table 2: Flow diagram suggestion**

*Table 2 could be more effectively presented as a flow diagram.*

**>> Thank you for this suggestion. We have considered presenting this as a flow diagram; however, given the multiple variables tracked at two timepoints (3 months and 12 months), we believe a revised table with clear annotation of new events provides the most complete and accessible presentation. We have added a footnote clarifying that the 12-month column represents new events between 3 and 12 months, and corrected the anticoagulation and catheter ablation figures as described above. We hope this revision addresses the concern adequately.**

**Journal Requirements**

**Requirement 1: Style requirements**

*Please ensure that your manuscript meets PLOS ONE's style requirements.*

**>> We have reviewed and ensured that the revised manuscript conforms to PLOS ONE's style requirements, including file naming conventions.**

**Requirement 2: Data Availability Statement**

*Please confirm whether your submission contains all raw data required to replicate the results of your study.*

**>> Due to ethical restrictions imposed by the institutional review board and the terms of participant consent, the individual-level data cannot be shared publicly. The data contain potentially identifiable information (age, sex, prefecture of residence, medical history) that could compromise participant privacy. Data requests may be directed to the Ethics Committee of Kyoto Prefectural University of Medicine. We have updated the Data Availability Statement accordingly.**

**Requirement 3: Recommended citations**

*If the reviewer comments include a recommendation to cite specific previously published works, please review and evaluate these publications.*

**>> We have reviewed the suggested references (VITAL-AF, GUARD-AF, and JAMA Cardiol 2021;6(5):558-567) and incorporated them where relevant in the Introduction section.**
