# Supplementary material for: Rates and predictors of hospital visits after “Possible AF” alerts from a home ECG monitor in older adults with hypertension: A sub-analysis of the Omron Heart Study
Source: PLoS One. 2026 Jul 17;21(7):e0353867. doi: 10.1371/journal.pone.0353867 (PMC13379017; doi:10.1371/journal.pone.0353867)
Supplement: Peer review history

# Author comment: Author response to Decision Letter 0

**DOI:** 10.1371/journal.pone.0353867.r001

**Transfer Alert**

This paper was transferred from another journal. As a result, its full editorial history (including decision letters, peer reviews and author responses) may not be present.

12 Dec 2025

# Peer review: Decision Letter 0

**DOI:** 10.1371/journal.pone.0353867.r002

**Authors:** Daniel Antwi-Amoabeng

23 Mar 2026

PONE-D-25-63371

Rates and Predictors of Hospital Visits After "Possible AF" Alerts from a Home ECG Monitor in Older Adults with Hypertension: A Sub-analysis of the Omron Heart Study

PLOS One

Dear Dr. kakei,

Thank you for submitting your manuscript to PLOS ONE. After careful consideration, we feel that it has merit but does not fully meet PLOS ONE’s publication criteria as it currently stands. Therefore, we invite you to submit a revised version of the manuscript that addresses the points raised during the review process.

==============================

Additional comments:

1. Only 220 of the 1,700 alerted participants had physician-adjudicated AF, meaning ~87% of "possible AF" alerts were not confirmed. Please integrate this context into the discussion and conclusions rather than attributing low follow-up rates primarily to patient-side barriers.

2. The 12-month column of Table 2 requires clarification as to whether reported events are cumulative since enrollment or incident between months 3-12; in particular the identical anticoagulation figures at both timepoints (18, 1.1%) despite additional Af diagnosis by 12 months requires explicit explanation.

3. The finding that 105 of 220 cardiologist-adjudicated AF cases received no clinical diagnosis within 12 months is perhaps the most consequential observation in the manuscript and deserves prominent discussion as a public health implication rather than a passing mention.

==============================

Please submit your revised manuscript by May 07 2026 11:59PM. If you will need more time than this to complete your revisions, please reply to this message or contact the journal office at plosone@plos.org. When you're ready to submit your revision, log on to https://www.editorialmanager.com/pone/ and select the 'Submissions Needing Revision' folder to locate your manuscript file.

Please include the following items when submitting your revised manuscript:

- A letter that responds to each point raised by the academic editor and reviewer(s). You should upload this letter as a separate file labeled 'Response to Reviewers'.
- A marked-up copy of your manuscript that highlights changes made to the original version. You should upload this as a separate file labeled 'Revised Manuscript with Track Changes'.
- An unmarked version of your revised paper without tracked changes. You should upload this as a separate file labeled 'Manuscript'.

If you would like to make changes to your financial disclosure, please include your updated statement in your cover letter. Guidelines for resubmitting your figure files are available below the reviewer comments at the end of this letter.

If applicable, we recommend that you deposit your laboratory protocols in protocols.io to enhance the reproducibility of your results. Protocols.io assigns your protocol its own identifier (DOI) so that it can be cited independently in the future. For instructions see: https://journals.plos.org/plosone/s/submission-guidelines#loc-laboratory-protocols. Additionally, PLOS ONE offers an option for publishing peer-reviewed Lab Protocol articles, which describe protocols hosted on protocols.io. Read more information on sharing protocols at https://plos.org/protocols?utm_medium=editorial-email&utm_source=authorletters&utm_campaign=protocols.

We look forward to receiving your revised manuscript.

Kind regards,

Daniel Antwi-Amoabeng, MD, MSc

Academic Editor

PLOS One

Journal Requirements:

When submitting your revision, we need you to address these additional requirements.

1. Please ensure that your manuscript meets PLOS ONE's style requirements, including those for file naming. The PLOS ONE style templates can be found at

https://journals.plos.org/plosone/s/file?id=wjVg/PLOSOne_formatting_sample_main_body.pdf and https://journals.plos.org/plosone/s/file?id=ba62/PLOSOne_formatting_sample_title_authors_affiliations.pdf

2. We note that your Data Availability Statement is currently as follows: All relevant data are within the manuscript and its Supporting Information files.

Please confirm at this time whether or not your submission contains all raw data required to replicate the results of your study. Authors must share the “minimal data set” for their submission. PLOS defines the minimal data set to consist of the data required to replicate all study findings reported in the article, as well as related metadata and methods (https://journals.plos.org/plosone/s/data-availability#loc-minimal-data-set-definition).

For example, authors should submit the following data:

- The values behind the means, standard deviations and other measures reported;

- The values used to build graphs;

- The points extracted from images for analysis.

Authors do not need to submit their entire data set if only a portion of the data was used in the reported study.

If your submission does not contain these data, please either upload them as Supporting Information files or deposit them to a stable, public repository and provide us with the relevant URLs, DOIs, or accession numbers. For a list of recommended repositories, please see https://journals.plos.org/plosone/s/recommended-repositories.

If there are ethical or legal restrictions on sharing a de-identified data set, please explain them in detail (e.g., data contain potentially sensitive information, data are owned by a third-party organization, etc.) and who has imposed them (e.g., an ethics committee). Please also provide contact information for a data access committee, ethics committee, or other institutional body to which data requests may be sent. If data are owned by a third party, please indicate how others may request data access.

3. If the reviewer comments include a recommendation to cite specific previously published works, please review and evaluate these publications to determine whether they are relevant and should be cited. There is no requirement to cite these works unless the editor has indicated otherwise.

Additional Editor Comments :

I recommend that the authors respond the the thorough comments by the reviewers to strengthen the work.

[Note: HTML markup is below. Please do not edit.]

Reviewers' comments:

Reviewer's Responses to Questions

**Comments to the Author**

1. Is the manuscript technically sound, and do the data support the conclusions?

The manuscript must describe a technically sound piece of scientific research with data that supports the conclusions. Experiments must have been conducted rigorously, with appropriate controls, replication, and sample sizes. The conclusions must be drawn appropriately based on the data presented.

Reviewer #1: Partly

Reviewer #2: Yes

Reviewer #3: Yes

**********

2. Has the statistical analysis been performed appropriately and rigorously?

Reviewer #1: Yes

Reviewer #2: N/A

Reviewer #3: Yes

**********

3. Have the authors made all data underlying the findings in their manuscript fully available?

The PLOS Data policy requires authors to make all data underlying the findings described in their manuscript fully available without restriction, with rare exception (please refer to the Data Availability Statement in the manuscript PDF file). The data should be provided as part of the manuscript or its supporting information, or deposited to a public repository. For example, in addition to summary statistics, the data points behind means, medians and variance measures should be available. If there are restrictions on publicly sharing data—e.g. participant privacy or use of data from a third party—those must be specified.

Reviewer #1: Yes

Reviewer #2: Yes

Reviewer #3: No

**********

4. Is the manuscript presented in an intelligible fashion and written in standard English?

PLOS ONE does not copyedit accepted manuscripts, so the language in submitted articles must be clear, correct, and unambiguous. Any typographical or grammatical errors should be corrected at revision, so please note any specific errors here.

Reviewer #1: Yes

Reviewer #2: Yes

Reviewer #3: Yes

**********

5. Review Comments to the Author

Please use the space provided to explain your answers to the questions above. You may also include additional comments for the author, including concerns about dual publication, research ethics, or publication ethics. (Please upload your review as an attachment if it exceeds 20,000 characters)

Reviewer #1: The manuscript offers a clinically important sub-analysis examining healthcare-seeking behaviors following "possible AF" alerts from home ECG monitoring devices. The study addresses a significant gap in digital health implementation by demonstrating low follow-up rates (23.5%) and identifying predictors of non-adherence. While the research question is relevant and the dataset substantial, several methodological concerns need to be addressed before publication.

Introduction:

It flows well and references relevant literature. There are however additional references that could be included here or in the discussion, such as the VITAL-AF (2024), GUARD-AF (2024), and JAMA Cardiol 2021;6;(5):558-567. doi:10.1001/jamacardio.2021.0038.

Methods and statistical analysis:

1. The authors need to describe the alerts shared with the subjects. Was there a role for the monitoring service? Did they receive in addition to the notification something that advises them to seek medical care? It is important to clarify these points.

2. What happened to subjects if they did not seek medical advice after an alert? Was there any specific reminder follow-up?

3. What happened with the 220 diagnosed with AF by physician confirmation? Were the subjects notified?

4. The analysis of time to seeking medical care is problematic given that some did not have accurate visit times. The authors should state explicitly whether dates of visits were missing for some or many participants (indicate the number), and how this impacts the Poisson model.

5. What were the results of the univariable analysis and what determined the inclusion of variables in the multivariable model? There are other variables that could be considered such as urban vs. rural residence, access to healthcare facilities, prior healthcare utilization patterns, etc. This may not be easy to have but just to acknowledge that they may have affected the results.

6. Why were the number of alerts and the display on 2 consecutive days not included in the multivariable model, but analyzed separately?

7. It helps to give the questionnaire as supplementary material or else to describe a bit the types of questions asked.

Results:

8. Better to provide subheadings for the results section.

9. The manuscript doesn't quantify what proportion came from app users vs. website volunteers.

10. There were additional 80 subjects who were diagnosed with AF. How were they discovered to have AF? It is unclear how many received anti-coagulation. It is indicated that only 18 received anticoagulation in total while at 3 months 18 were prescribed AC. With the additional 80 after 3 months, none was newly prescribed an AC?

11. In follow-up to above, Table 2 is confusing: The 12-month column shows cumulative events since enrollment, not new events between 3-12 months. The presentation makes it appear that only 80 new AF diagnoses occurred between 3-12 months, but it's unclear if this includes the 35 from the 3-month survey.

12. When describing predictors, clarifying the clinical effect size would improve readability (e.g., “participants without palpitations were ~50% less likely to follow up”).

13. Of 220 with confirmed AF, only 115 (52.3%) were diagnosed clinically within 12 months; what happened to the other 105?

Discussion:

14. The paper should clarify how often alerts were false positives, whether false-positive alerts contributed to avoidance or skepticism, and that behavioral analysis is on alerts, not confirmed AF episodes. This distinction is critical for clinicians and researchers.

15. Only 12.9% of "possible AF" alerts were confirmed. This 87% false-positive rate is a critical context for understanding low follow-up but is barely discussed. The discussion focuses on patient barriers without adequately addressing whether low follow-up might be partially rational given high false-positive rates.

16. The inverse association between alcohol consumption and healthcare utilization is interesting but deserves deeper consideration. Is this confounded by unmeasured socioeconomic factors? Could alcohol consumption be a marker for lower health anxiety?

17. Limitations section misses important considerations:

a. No validation of self-reported hospital visits against medical records

b. No information on device accuracy/false-positive rate in this specific population

c. No data on reasons participants chose not to seek care

d. No assessment of healthcare system factors (appointment availability, cost barriers)

Reviewer #2: The Manuscript titled Rates and Predictors of Hospital Visits After "Possible AF" Alerts from a Home ECG

Monitor in Older Adults with Hypertension: A Sub-analysis of the Omron Heart Study; is technically sound and the data support the conclusion. The manuscript is written in standard English and feels smooth while going through.

Reviewer #3: The authors present a sub-analysis of the Omron Heart Study examining healthcare-seeking behavior after ‘possible AF’ alerts from a home ECG device in older adults with hypertension. The topic is relevant given the increasing use of digital AF screening tools, as well as emerging evidence supporting earlier AF treatment.

Overall, the manuscript is well structured, and the finding of low follow-up rates after alerts is noteworthy. However, several aspects of the manuscript would benefit from improved framing. In particular, the conclusions should be more guarded, as the reasons underlying low healthcare-seeking behavior were not explored in this study.

Minor comments:

Line 46 - the authors state that these home devices have high accuracy in AF detection. What is the basis for this statement? In this study, only a minority of device-triggered alerts were ultimately adjudicated as AF, highlighting that alerts represent possible rather than confirmed AF. The wording should be adjusted to reflect this distinction.

Methods:

The authors should briefly describe the larger Omron heart study to provide some context.

It would be helpful to explicitly state that participants were instructed to record ECGs twice daily for 3 months, as this is not immediately clear from the current description.

Results:

Among the 1,682 respondents, how many had at least one AF alert? This would help contextualize the denominator for subsequent analyses.

Similarly, among the 220 participants with physician-adjudicated AF, how many responded to the follow-up questionnaire? This is important for interpreting the findings related to diagnosis and healthcare utilization.

Discussion:

The extrapolation to nationwide estimates of undiagnosed AF is an overreach. These estimates are based on a relatively small subgroup (220 adjudicated AF cases, with only 35 new diagnoses) and are not directly aligned with the primary objective of the study. I would recommend removing or substantially tempering this section.

The interpretation of ‘low follow-up rates’ appears to be somewhat one-sided. A ‘possible AF’ alert from a home device does not represent a confirmed diagnosis. In many cases, deferring immediate medical evaluation may be reasonable, particularly if they have only had 1 alert, for example, or overall risk is low. The authors should consider presenting this finding more neutrally and acknowledge that some degree of non-follow-up may reflect reasonable decision-making

Table 2 could be more effectively presented as a flow diagram

**********

6. PLOS authors have the option to publish the peer review history of their article (what does this mean?). If published, this will include your full peer review and any attached files.

If you choose “no”, your identity will remain anonymous but your review may still be made public.

**Do you want your identity to be public for this peer review?**  For information about this choice, including consent withdrawal, please see our Privacy Policy.

Reviewer #1: No

Reviewer #2: No

Reviewer #3: No

**********

[NOTE: If reviewer comments were submitted as an attachment file, they will be attached to this email and accessible via the submission site. Please log into your account, locate the manuscript record, and check for the action link "View Attachments". If this link does not appear, there are no attachment files.]

To ensure your figures meet our technical requirements, please review our figure guidelines: https://journals.plos.org/plosone/s/figures

You may also use PLOS’s free figure tool, NAAS, to help you prepare publication quality figures: https://journals.plos.org/plosone/s/figures#loc-tools-for-figure-preparation.

NAAS will assess whether your figures meet our technical requirements by comparing each figure against our figure specifications.

# Author comment: Author response to Decision Letter 1

**DOI:** 10.1371/journal.pone.0353867.r003

4 May 2026

We have addressed all reviewer and editor comments in the attached Response to Reviewers document.

Attachment Submitted filename: Response_to_Reviewers_PONE-D-25-63371_v2.docx

# Peer review: Decision Letter 1

**DOI:** 10.1371/journal.pone.0353867.r004

**Authors:** Daniel Antwi-Amoabeng, Daniel Antwi-Amoabeng

1 Jul 2026

Rates and Predictors of Hospital Visits After "Possible AF" Alerts from a Home ECG Monitor in Older Adults with Hypertension: A Sub-analysis of the Omron Heart Study

PONE-D-25-63371R1

Dear Dr. Senoo,

We’re pleased to inform you that your manuscript has been judged scientifically suitable for publication and will be formally accepted for publication once it meets all outstanding technical requirements.

Within one week, you’ll receive an e-mail detailing the required amendments. When these have been addressed, you’ll receive a formal acceptance letter and your manuscript will be scheduled for publication.

An invoice will be generated when your article is formally accepted. Please note, if your institution has a publishing partnership with PLOS and your article meets the relevant criteria, all or part of your publication costs will be covered. Please make sure your user information is up-to-date by logging into Editorial Manager at Editorial Manager® and clicking the ‘Update My Information' link at the top of the page. For questions related to billing, please contact billing support.

If your institution or institutions have a press office, please notify them about your upcoming paper to help maximize its impact. If they’ll be preparing press materials, please inform our press team as soon as possible -- no later than 48 hours after receiving the formal acceptance. Your manuscript will remain under strict press embargo until 2 pm Eastern Time on the date of publication. For more information, please contact onepress@plos.org.

Kind regards,

Daniel Antwi-Amoabeng, MD, MSc

Academic Editor

PLOS One

Additional Editor Comments (optional):

Reviewers' comments:

Reviewer's Responses to Questions

**Comments to the Author**

1. If the authors have adequately addressed your comments raised in a previous round of review and you feel that this manuscript is now acceptable for publication, you may indicate that here to bypass the “Comments to the Author” section, enter your conflict of interest statement in the “Confidential to Editor” section, and submit your "Accept" recommendation.

Reviewer #1: All comments have been addressed

**********

2. Is the manuscript technically sound, and do the data support the conclusions?

The manuscript must describe a technically sound piece of scientific research with data that supports the conclusions. Experiments must have been conducted rigorously, with appropriate controls, replication, and sample sizes. The conclusions must be drawn appropriately based on the data presented.

Reviewer #1: Yes

**********

3. Has the statistical analysis been performed appropriately and rigorously?

Reviewer #1: Yes

**********

4. Have the authors made all data underlying the findings in their manuscript fully available?

The PLOS Data policy requires authors to make all data underlying the findings described in their manuscript fully available without restriction, with rare exception (please refer to the Data Availability Statement in the manuscript PDF file). The data should be provided as part of the manuscript or its supporting information, or deposited to a public repository. For example, in addition to summary statistics, the data points behind means, medians and variance measures should be available. If there are restrictions on publicly sharing data—e.g. participant privacy or use of data from a third party—those must be specified.

Reviewer #1: Yes

**********

5. Is the manuscript presented in an intelligible fashion and written in standard English?

PLOS ONE does not copyedit accepted manuscripts, so the language in submitted articles must be clear, correct, and unambiguous. Any typographical or grammatical errors should be corrected at revision, so please note any specific errors here.

Reviewer #1: Yes

**********

6. Review Comments to the Author

Please use the space provided to explain your answers to the questions above. You may also include additional comments for the author, including concerns about dual publication, research ethics, or publication ethics. (Please upload your review as an attachment if it exceeds 20,000 characters)

Reviewer #1: I have carefully reviewed the authors' point-by-point response and the revised manuscript. Overall, I am satisfied that the authors have addressed my concerns in a thorough manner. The manuscript is substantially improved and I believe it is now suitable for publication.

**********

7. PLOS authors have the option to publish the peer review history of their article (what does this mean?). If published, this will include your full peer review and any attached files.

If you choose “no”, your identity will remain anonymous but your review may still be made public.

**Do you want your identity to be public for this peer review?**  For information about this choice, including consent withdrawal, please see our Privacy Policy.

Reviewer #1: No

**********

# Editor report: Acceptance letter

**DOI:** 10.1371/journal.pone.0353867.r005

**Authors:** Daniel Antwi-Amoabeng, Daniel Antwi-Amoabeng

PONE-D-25-63371R1

PLOS One

Dear Dr. Senoo,

I'm pleased to inform you that your manuscript has been deemed suitable for publication in PLOS One. Congratulations! Your manuscript is now being handed over to our production team.

At this stage, our production department will prepare your paper for publication. This includes ensuring the following:

* All references, tables, and figures are properly cited

* All relevant supporting information is included in the manuscript submission,

* There are no issues that prevent the paper from being properly typeset

You will receive further instructions from the production team, including instructions on how to review your proof when it is ready. Please keep in mind that we are working through a large volume of accepted articles, so please give us a few days to review your paper and let you know the next and final steps.

Lastly, if your institution or institutions have a press office, please let them know about your upcoming paper now to help maximize its impact. If they'll be preparing press materials, please inform our press team within the next 48 hours. Your manuscript will remain under strict press embargo until 2 pm Eastern Time on the date of publication. For more information, please contact onepress@plos.org.

You will receive an invoice from PLOS for your publication fee after your manuscript has reached the completed accept phase. If you receive an email requesting payment before acceptance or for any other service, this may be a phishing scheme. Learn how to identify phishing emails and protect your accounts at https://explore.plos.org/phishing.

If we can help with anything else, please email us at customercare@plos.org.

Thank you for submitting your work to PLOS ONE and supporting open access.

Kind regards,

PLOS ONE Editorial Office Staff

on behalf of

Dr. Daniel Antwi-Amoabeng

Academic Editor

PLOS One
